# Acoustic emission characteristics and damage evolution of basalt by microwave irradiation

**DOI:** 10.1038/s41598-023-30220-y

**Published:** 2023-04-12

**Authors:** Jinqiang Yang, Chaolin Wang, Yu Zhao, Jing Bi

**Affiliations:** 1 College of Civil Engineering, Guizhou University, Huaxi District, Guiyang, 550025 Guizhou China; 2 Engineering Laboratory for Safe and Precise Coal Mining of Anhui Province, Anhui University of Science and Technology, Huainan, 232001 China

**Keywords:** Civil engineering, Mineralogy

## Abstract

The microwave-assisted rock breaking technology has been proven to be feasible, and has received considerable attention in the field of civil and mining engineering. A copper foil was used to wrap basalt to simulate rock excavation of practical application scenario in this paper. To this end, a multi-mode cavity with an operating frequency of 2.45 GHz was used to conduct microwave irradiation experiments on basalts with different irradiation times and different power. The thermal properties, AE characteristics, and damage evolution process of basalt were studied. The results show that the high heat generated by microwave leads to the development of cracks in the upper part of basalt. The higher the power level, the higher the degree of crack propagation in the sample, the lower the basalt strength, and the more active the AE activity. The fluctuation rule of the b value indicates that the basalt is dominated by small-scale microfractures before failure. High power levels or long irradiation time lead to more microwave-induced cracks participating in the failure process during loading. Compared with unheated basalt, microwave-heating basalt detects the characteristics of the precursor of failure in advance. The AE source location and the nephogram of the maximum principal stress of microwave-treated basalt reflected that the fracture path begins in the upper part of the rock. In addition, the combination of high power level and short irradiation time can achieve the purpose of energy saving.

## Introduction

In the mining and tunneling projects, hard rock breakage is an urgent problem to be solved when crossing or being in rock stratum. The technology of microwave-assisted rock breaking is a promising method for rock breaking, because of its high efficiency, sustainability, and less pollution. Theoretical and experimental research shows that the technology of microwave-induced fracturing can improve efficiency and reduce energy consumption in practical engineering^1–5^.

The principle of microwave working is dielectric heating, in which polar molecules in alternating electric fields convert microwave energy into heat through rapid rotation^6^. The collisions and friction between molecules generate a lot of heat, causing the temperature increase of material heated^7^. The minerals of rocks have different microwave absorption capacities^8–10^. Microwaves selectively heat minerals with high microwave absorption capacity, resulting in thermal mismatch stress and thermal gradient stress within the material^11,12^. Thermal mismatch stress is the result of different thermal expansion coefficients of minerals and thermal gradient stress is caused by the temperature difference between different phases. Internal mechanical stresses are sufficient to induce intergranular and transgranular cracks in the grain matrix under microwave heating^13–16^.

Acoustic emission (AE) can monitor the development and evolution of microcracks in the rock^17,18^. The b value of AE can afford early warnings of imminent rupture^19–22^. The JCMS fracture classification method is usually used to explore the fracturing type during the failure process^23,24^. The approach distinguishes between tensile and shear fracturing modes by computing maximum amplitude (RA = rise time/maximum amplitude) and average frequency (AF = AE count/duration)^25,26^. The AE signals with high AF and low RA values indicate the generation of tension crack, and high RA and low AF values generally indicate the generation of shear crack. Hence, it is practicable to analyze the initiation, development, and penetration evolvement mechanism of microcracks under different time and power terms by analyzing the b-value and RA-AF^27^.

The technology of microwave-assisted rock breaking was first proposed by Gushchin et al. and Gushchin et al. in 1973^28,29^. Their research showed that microwaves were effective for hard rock breaking. Therefore, when the microwave was used in combination with the tunnel boring machines, the efficiency of rock breaking was greatly improved. Ferri Hassani et al.^30^ studied the effect of microwave radiation on three types of hard rocks: norite, granite, and basalt. The results showed that the tensile strength and uniaxial compressive strength decrease with exposure time and power. Hartlieb et al.^31^ used a 24 kW open-ended microwave system to heat the granite. The results showed a distinct fracture network appears throughout the rock sample surface at a depth of about 100 mm, and the force required to cut the rock is significantly reduced. Zheng et al.^11^ used an open-ended microwave system to treat three types of igneous rocks (gabbro, monzonite, and granite). The three types of igneous rocks treated were weakened to different degrees, and cracks or melting/fracturing occurred, respectively. Yao et al.^32^ simulated the microwave treatment before excavation and studied the thermal properties and structural degradation of the sandstone samples. The results showed that the number of macropores and mesopores increased after microwave treatment.

The intensity of the microwave decays exponentially as the microwave penetrates the material. The decay rate of microwave depends on the frequency of the electromagnetic wave and the dielectric constant of the material. The penetration depth is the distance that the energy density of microwave decays to 1/e (e = 2. 718 as the Euler’s number) of their initial energy density^33^. The theoretical penetration depth of microwaves is expressed as^33^:

1 $$z=\frac{{\lambda }_{0}\pm \sqrt{{\varepsilon }^{^{\prime}}}}{2\pi {\varepsilon }^{"}}$$

where $\mathrm{z}$ is the penetration depth (m), ${\lambda }_{0}$ is the wavelength of the appropriate frequency (m), ${\varepsilon }^{\mathrm{^{\prime}}}$ is the dielectric constant of the material, and ${\varepsilon }^{"}$ is the loss factor of the material.

When exposed to a microwave emitted from an open waveguide, the power density of the electromagnetic energy and the electric field in the material decreases exponentially as follows^6^:

2 $$P(z)={P}_{0}{\mathrm{e}}^{-z/{z}_{0}}$$

where $P(z)$ is the power density at depth z, ${P}_{0}$ is the incident power density, and z0 is the depth at which the power density magnitude decays to 1/e of its value at the surface.

Limited by the test conditions, this paper adopts the copper foil wrapping method^32^ to pretreat the rock to better study the influence of microwave irradiation on the rock during the excavation process. Microwave irradiation experiments were conducted on basalt with different exposure times and different microwave power levels. Then, the surface temperature of the samples was measured by infrared technology to study the surface temperature distribution after microwave irradiation. Combined with the corresponding AE and DIC techniques, the damage evolution process of treated specimens under uniaxial compression is analyzed.

## Methodology and materials

### Rock material and specimen preparation

The basalt from the Chifeng Region of China was selected for the microwave irradiating test in this study. The mineral composition mainly consists of plagioclases, pyroxene, olivine, metallic minerals, and other substrates^34^. The cores taken from the same rock were processed into standard specimens 100 mm high and 50 mm diameter. During the preparation process, the non-parallelism and non-perpendicularity of the basalt samples were controlled below 0. 02 mm. The samples were dried in an oven for 24 h at 105 ℃ to avoid the influence of moisture on the test results before microwave heating^35^.

**Figure 1:**
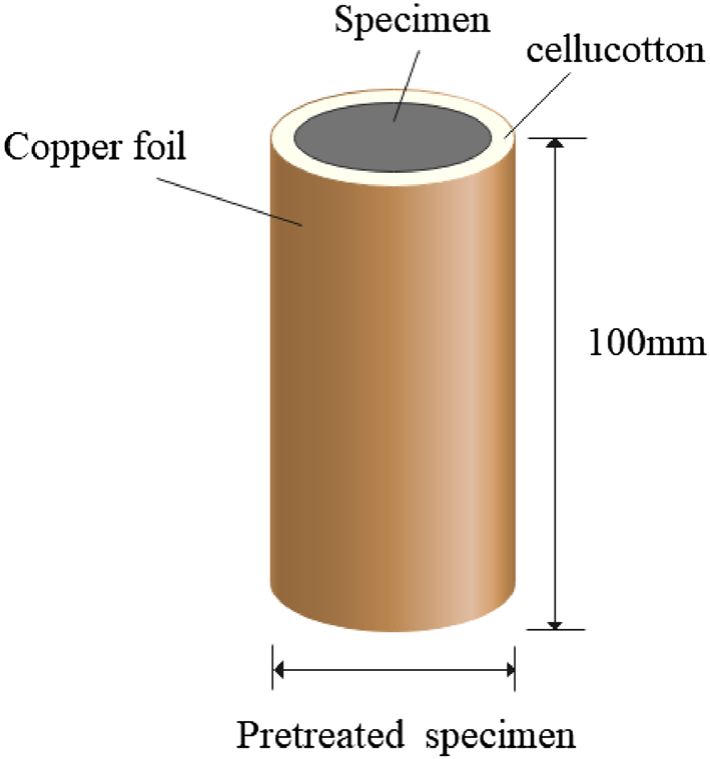
Preparation of specimen.

During rock excavation projects, only one rock face is microwave irradiated. In previous microwave tests, all the surfaces of the tested rock sample absorb microwave energy. This is not consistent with practical engineering conditions^3,32^. Therefore, we used the copper-foil-wrapping method^32^ to pre-treated the samples to simulate rock excavation of the actual field conditions with microwave irradiation. To study the damage of samples, the basalt was wrapped with ceramic fiber cotton and coppered foil with a thickness of 10 mm and 0.1 mm respectively (as shown in Fig. 1).

### Testing equipment

1. Multimode microwave system

**Figure 2:**
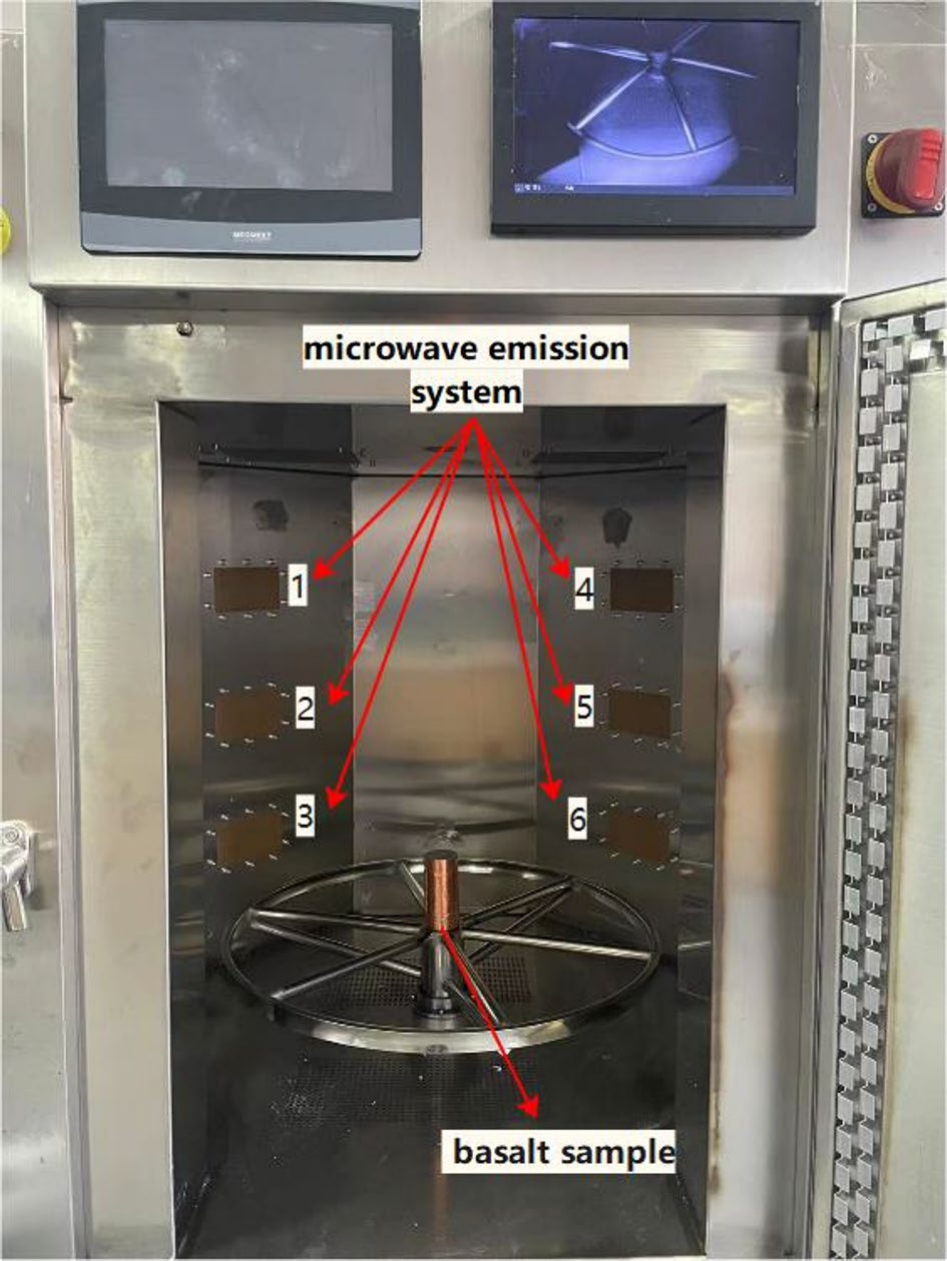
Multimode microwave system.

A multimode microwave system with a microwave power range of 1.5–5.1 kW was used in this experiment. The microwave emission system is equipped with six air-cooled magnetrons with a maximum output power of 5.1 kW and a working frequency of 2.45 GHz. The microwave emission systems are evenly distributed in the cavity, as shown in Fig. 2.

2. Thermal imaging test

An industrial thermal imager (ITI) apparatus was used for the thermal imaging of the samples. The infrared resolution ratio is 382 × 288 pixels. The target temperature ranged from − 20 to 900 °C with a precision of 2%, and the thermal sensitivity (the noise equivalent temperature difference) is less than 0.02 °C. The spectral range is from 7.5 to 14 μm. The imaging frequency is 80 Hz.

3. Uniaxial compression test

**Figure 3:**
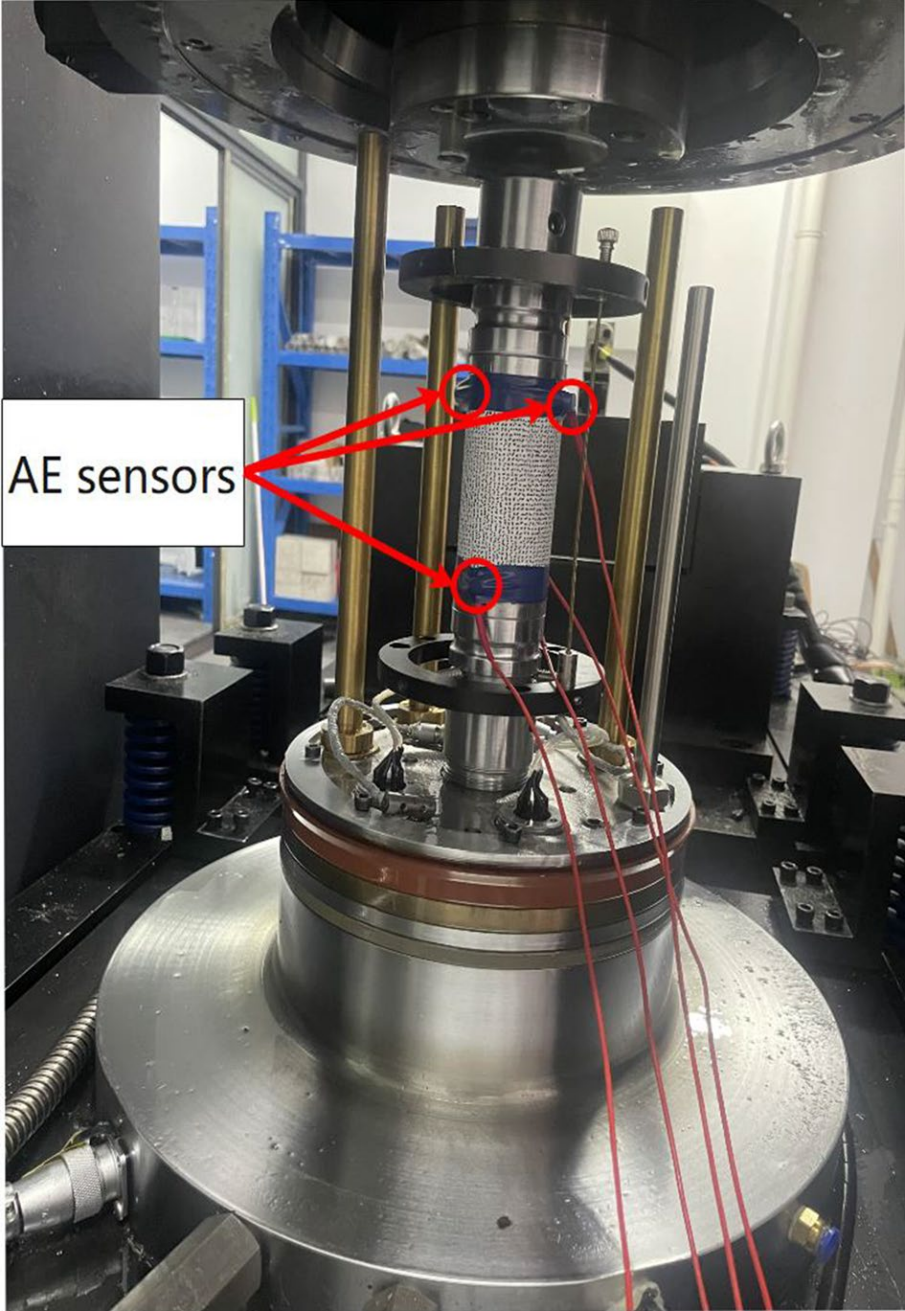
Uniaxial compression test and AE test.

After the microwave tests, uniaxial compression tests were carried out on these treated basalt specimens through rock mechanical testing system, as shown in Fig. 3. The specimen was loaded to failure at a displacement rate of 0.1 mm/min.

4. AE system

The PCI-2 AE detection system manufactured by American Physical Acoustics Corporation (PAC) is used for AE experiments. In the test, the sampling frequency was set at 5 MHz, and the threshold value was set at 40 dB. Three AE sensors were attached to the surface of the sample with Vaseline and secured with tape, as shown in Fig. 3.

5. DIC system

The DIC system is an optical measurement technique for measuring the deformation of rock. The system includes an adjustable digital camera, an image acquisition control box, and a high-performance computer.

### Experimental methods

The microwave irradiation test is performed at different power levels and treated times. Four specimens were microwave irradiated with the same treating time, i.e. at a power of 1.5 kW, 2.7 kW, 3.9 kW, and 5.1 kW for 10 min, respectively. The other specimens were microwave irradiated at the same microwave energy. Specifically, microwave radiation was conducted on samples for 34 min, 18.88 min, 13.08 min, and 10 min at power of 1.5 kW, 2.7 kW, 3.9 kW, and 5.1 kW, respectively. In addition, three untreated samples were set as a comparison group. Table 1 lists the information of the tested sample.

**Table. 1:** Microwave irradiated project.

Sample no	Microwave radiation	Remark
I	1.5 kw–10 min	Microwave irradiated at 1.5 kW for 10 min
II	2.7 kw–10 min	Microwave irradiated at 2.7 kW for 10 min
III	3.9 kw–10 min	Microwave irradiated at 3.9 kW for 10 min
IV	1.5 kw–34 min	Microwave irradiated at 1.5 kW for 34 min
V	2.7 kw–18.88 min	Microwave irradiated at 2.7 kW for 18.88 min
VI	3.9 kw–13.08 min	Microwave irradiated at 3.9 kW for 13.08 min
VII	5.1 kw–10 min	Microwave irradiated at 5.1 kW for 10 min

**Figure 4:**
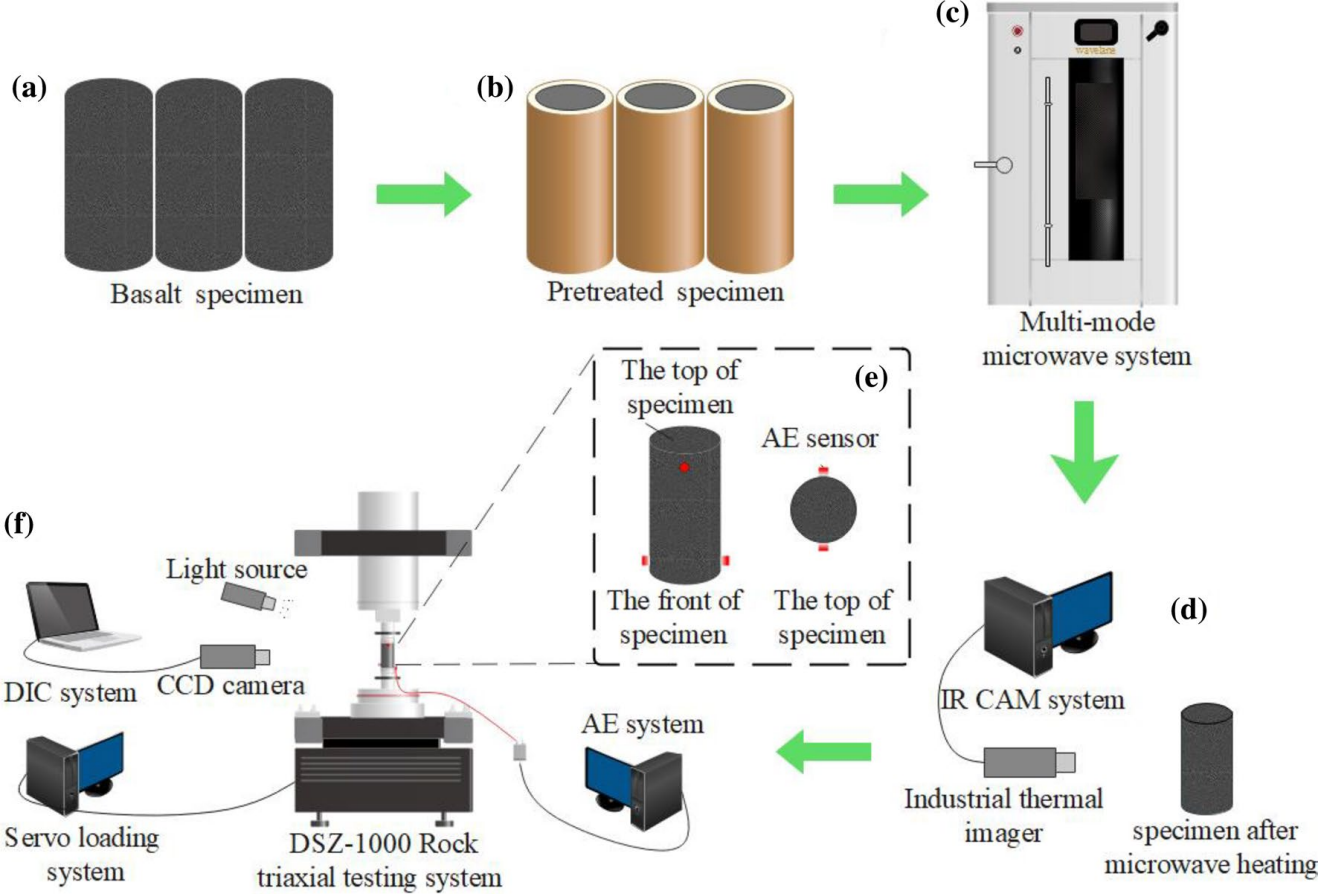
The main equipment and test flow chart. (**a**) Basalt specimen; (**b**) pretreated specimen; (**c**) multi-mode microwave system; (**d**) thermal imaging test; (**e**) arrangement of AE sensors; (**f**) uniaxial compressive testing system.

Figure 4 shows the experimental flow chart of this study. The samples were respectively subjected to thermal imaging test, uniaxial test, AE test, and DIC test after microwave test.

## Results

### Temperature curve

**Figure 5:**
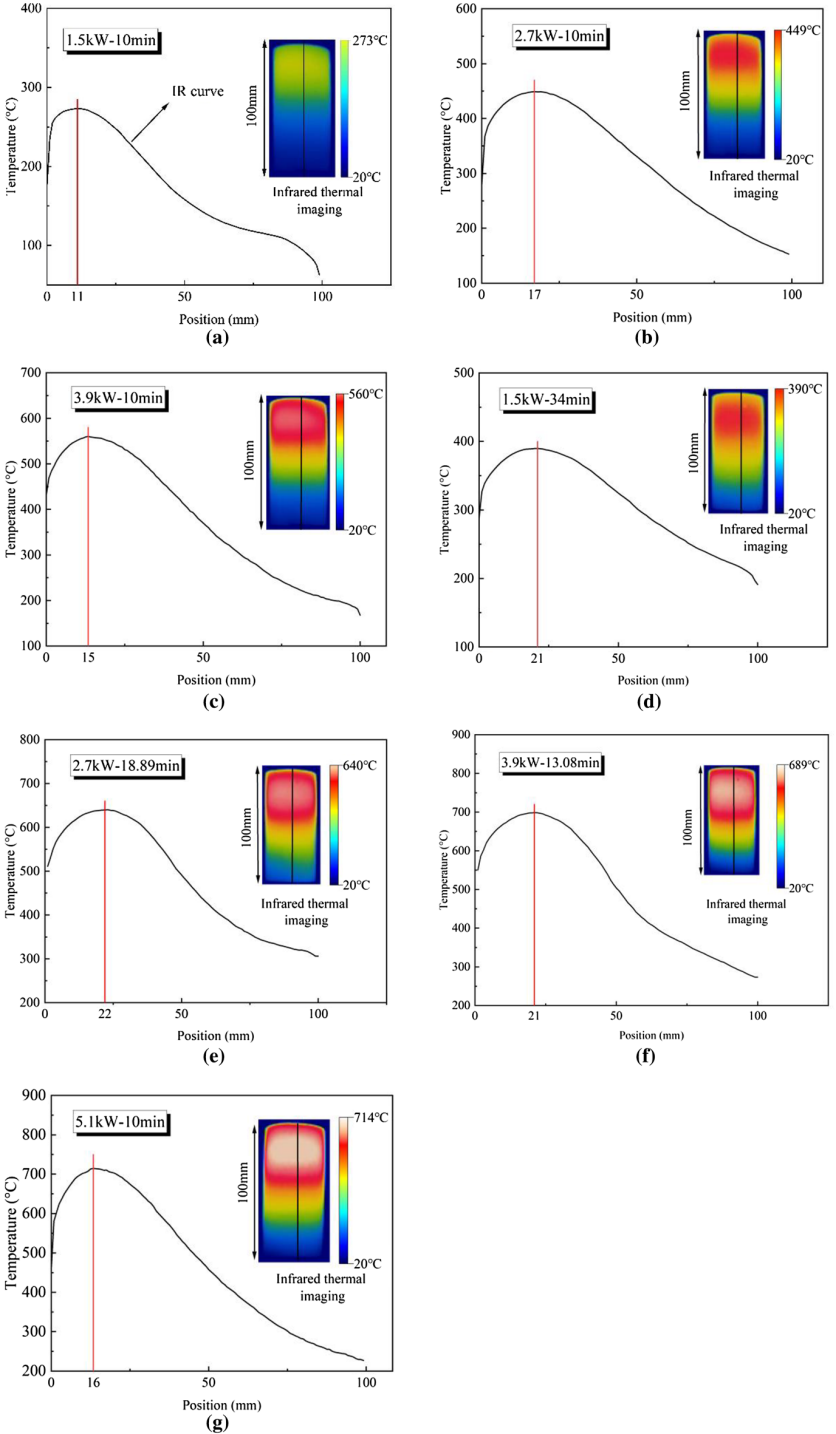
Temperature distribution along the vertical axis of specimens heated for basalt (red solid line represents the maximum temperature): (**a**) at 1.5kW for 10 min; (**b**) at 2.7kW for 10 min; (**c**) at 3.9 kW for 10 min; (**d**) at 1.5kW for 34 min; (**e**) at 2.7 kW for 18.88 min; (**f**) at 3.9 kW for 13.08 min; (**g**) at 5.1 kW for 10 min.

The microwave penetration depth is independent of the power level (Eq. 1), while the microwave energy absorbed by the dielectric material causes the temperature to rise and decays exponentially with increasing depth (Eq. 2). Figure 3 shows the temperature distribution of the central axis (the solid black line of infrared thermal imaging in Fig. 5) of the microwave irradiated specimens.

As shown in the Fig. 5, the shape of the infrared ray (IR) curve under different microwave treatment conditions shows a first increase and then decrease with the depth. The temperature rise of the sample is related to the mineral content of the sample (e.g., feldspar, pyroxene, olivine, hornblende, biotite, etc.) and the heat conduction between the particles. Pyroxenes, is sensitive to microwave, which exhibits significant heating effects under microwave radiation. Olivines exhibit significant thermal expansion^35^. The thermal expansion of olivine causes thermal stress, resulting in the formation of microcracks in the rock. As the microwave irradiation time increases, the microcracks slip and interconnect to form penetration cracks. After microwave treatment, the temperature of the top surface is less than the maximum surface temperature, which is due to the heat within the basalt being transferred to the atmosphere by blackbody radiation and air convection. And the surface temperature away from the top surface decreases rapidly, which is the result of insufficient heat transfer due to the decrease of thermal conductivity of the basalt with the increase in temperature^36^. The highest surface temperature of the basalt is found approximately 2 cm from the top surface^37^.

**Figure 6:**
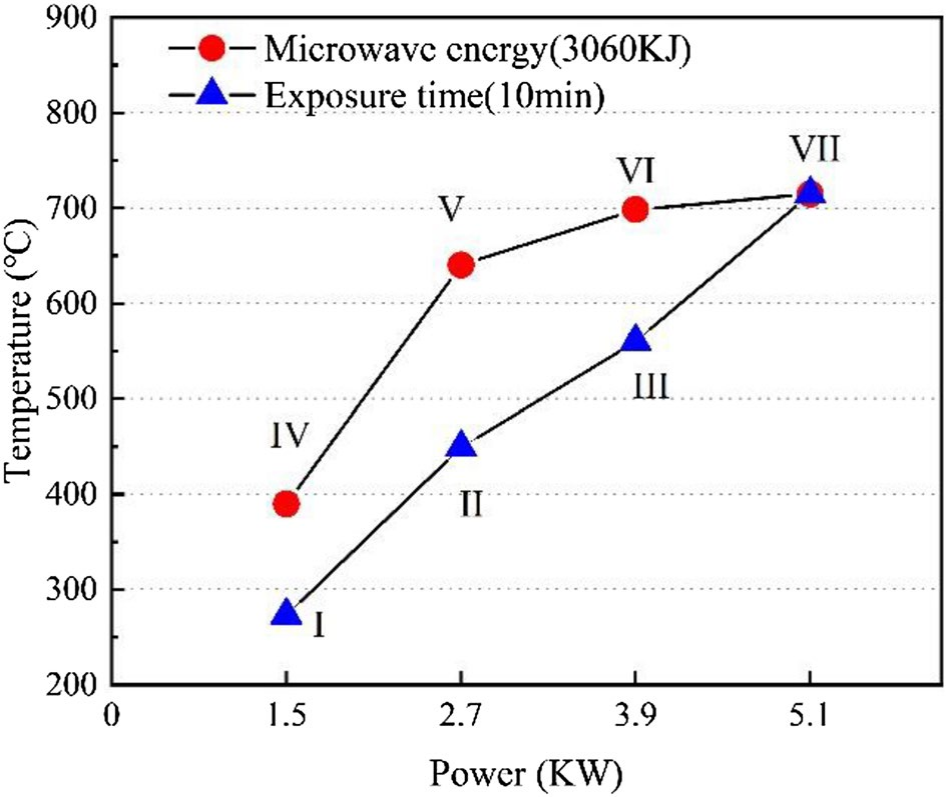
Relationship between the maximum surface temperature with power level.

The relationship between the highest temperature and power level of the surface of basalt under microwave irradiation is shown in Fig. 6. The highest temperature of the surface of basalt samples increased with power. At the same power level, the maximum surface temperature of the basalt sample is higher with the longer microwave irradiation time. Specifically, the highest surface temperature of samples after 10 min of microwave irradiation at the power of 1.5 kW, 2.7 kW, 3.9 kW, and 5.1 kW is 273 ℃, 449 ℃, 560 ℃, and 714 ℃, respectively. Under the same microwave energy (3060 kJ), the highest temperatures of the surface of basalt samples at the power of 1.5 kW, 2.7 kW, 3.9 kW, and 5.1 kW are 390 ℃, 640 ℃, 689 ℃, and 714 ℃, respectively.

### Heating effect

**Figure 7:**
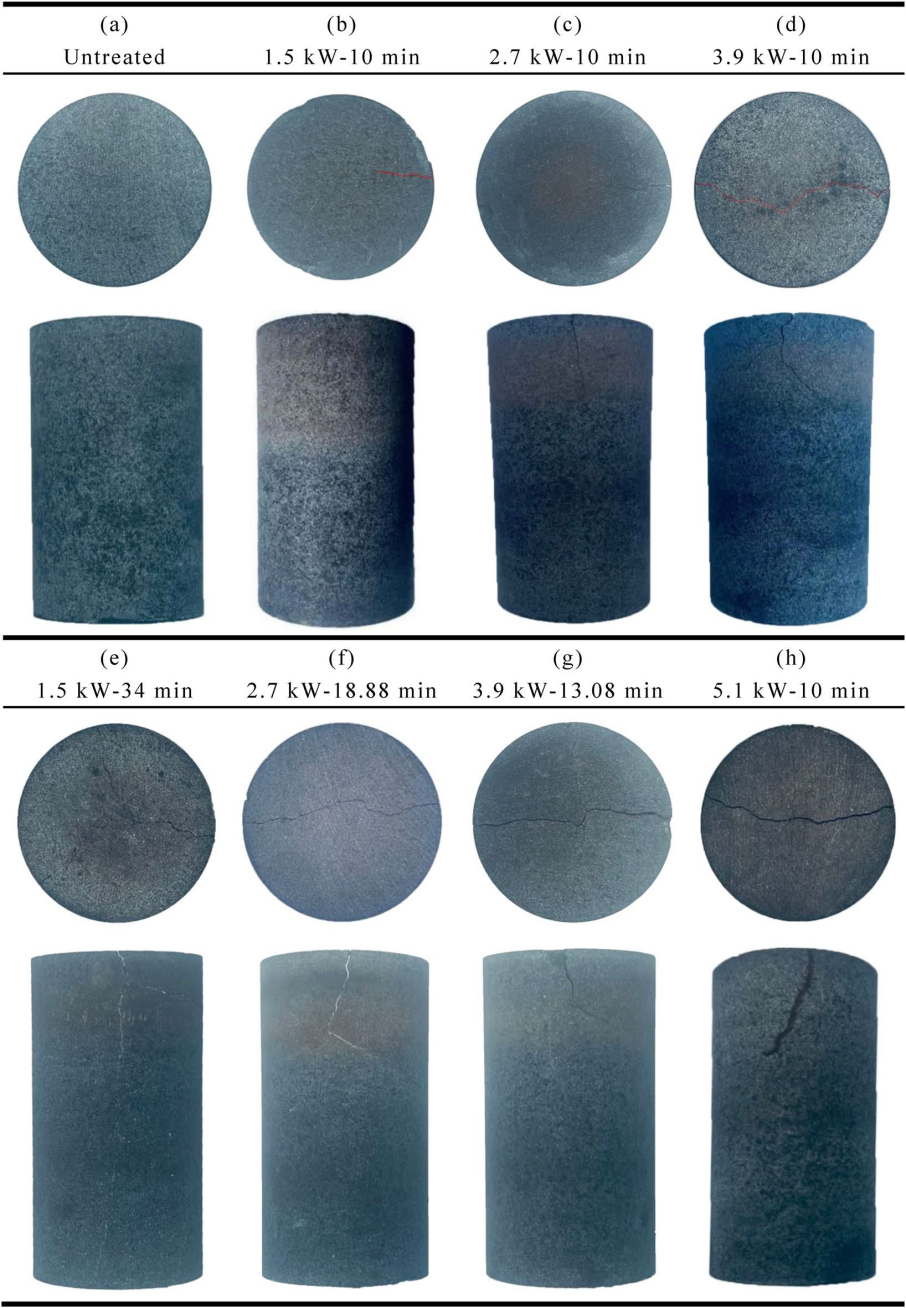
Basalt samples after microwave radiation: (**a**) Untreated; (**b**) at 1.5kW for 10 min; (**c**) at 2.7kW for 10 min; (**d**) at 3.9 kW for 10 min; (**e**) at 1.5kW for 34 min; (**f**) at 2.7 kW for 18.88 min; (**g**) at 3.9 kW for 13.08 min; (**h**) at 5.1 kW for 10 min.

Figure 7 shows the basalt sample after microwave treatment. After microwave treatment, macroscopic cracks can be seen on the top surface and cylinder surface of basalt samples. The extent of cracking development depends on the applied power level and exposure time.

When the power level is low (1.5 kW), fewer cracks are visible on the surface of basalt samples, as shown in Fig. 7b. Under the same microwave energy (3060 kJ), with the increase of the power level, the crack width of the top surface of basalt samples increases significantly. It can be found that the microwave-treated basalt samples with high-power short-time (see Fig. 7h) show more obvious damage than that of samples treated with low-power long-time (Fig. 7e). This indicates that high-power microwave irradiation has a significant weakening effect on the basalt sample. With the same power level, by comparing with Fig. 7d,g, when the irradiation time increases from 10 to 13.08 min, the maximum temperature of the basalt sample increases from 560 to 689 ℃, and the crack width becomes larger obviously.

### Mechanical properties

**Figure 8:**
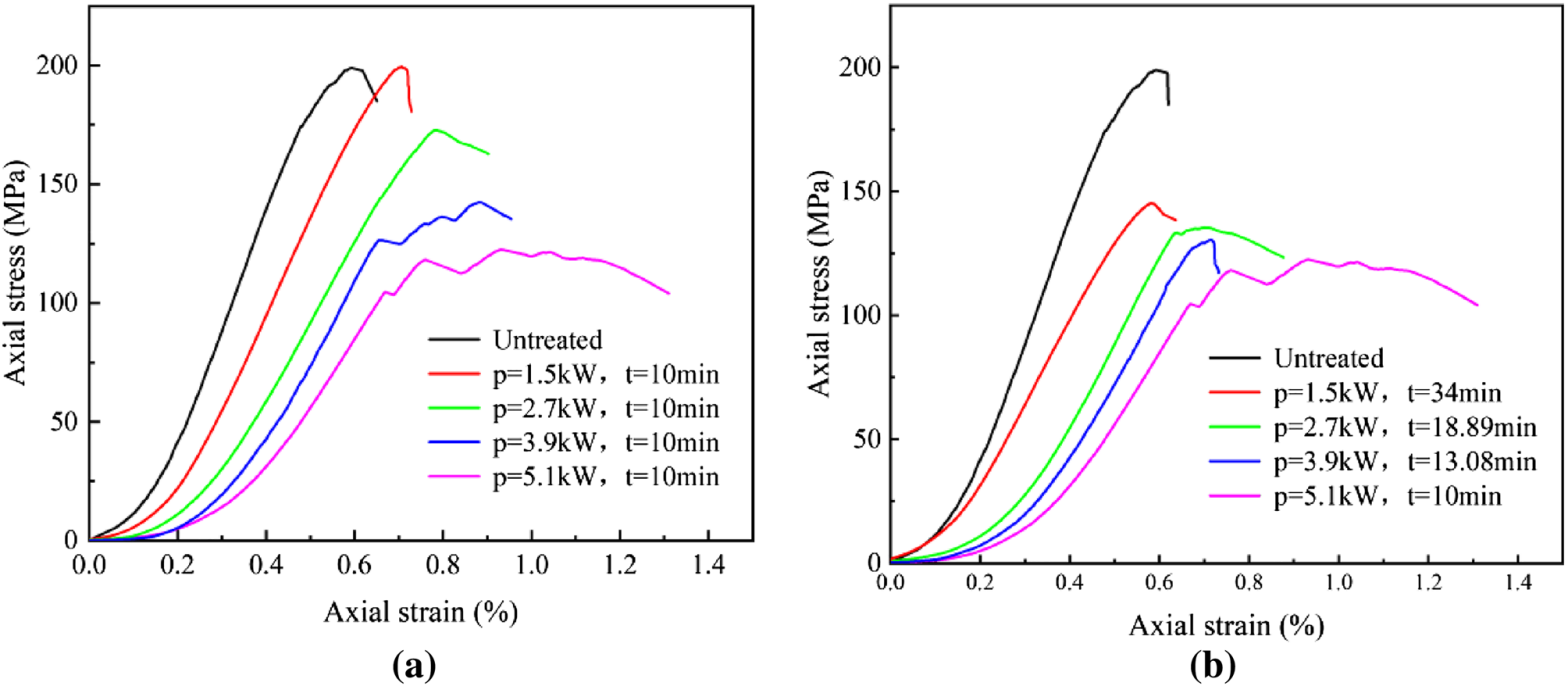
The stress–strain curves of treated basalt under uniaxial compression. (**a**) Microwave irradiation time t = 10 min; (**b**) Microwave energy 3060 kJ.

The representative stress–strain of basalt specimens under uniaxial compression after microwave radiation is shown in Fig. 8. The stress–strain curve of microwave-treated basalt goes through four stages: a crack closure stage, an elastic deformation stage, a crack stable propagation stage, and a post-peak deformation stage^38–40^. During the crack closure stage, the pre-existing microcracks inside rocks gradually close, resulting in a nonlinear deformation of the stress–strain curve of the specimen. With the increase of axial stress, the stress–strain curve enters the linear elastic stage. Subsequently, when the axial stress–strain curve enters the stage of crack stable propagation, the microcracks are induced and developed in the rock until the peak strength is reached and finally enters the post-peak deformation stage.

The nonlinear characteristics of the stress–strain curve in the crack closure stage gradually decrease with the increase of power level in Fig. 8. In the line elastic deformation stage, the stress–strain curve shows obvious linear elastic characteristics, and the slope of the curve decreases with the increase in power level. Correspondingly, the crack stable propagation stage is prolonged, plasticity is enhanced and the peak axial strain increases. As the microwave power level increases, the curve of the crack stable propagation stage gradually becomes flat. The phenomena imply that as the microwave power level increases, more and more microcracks and fractures are produced inside the rock. When the axial stress reaches its peak, the strain increases sharply and the axial stress decreases rapidly, indicating that the specimen has lost its load-bearing capacity. Thus, the stress–strain curve of the basalt gradually shows a strain-softening behavior with the increase in power level ^41–43^.

**Figure 9:**
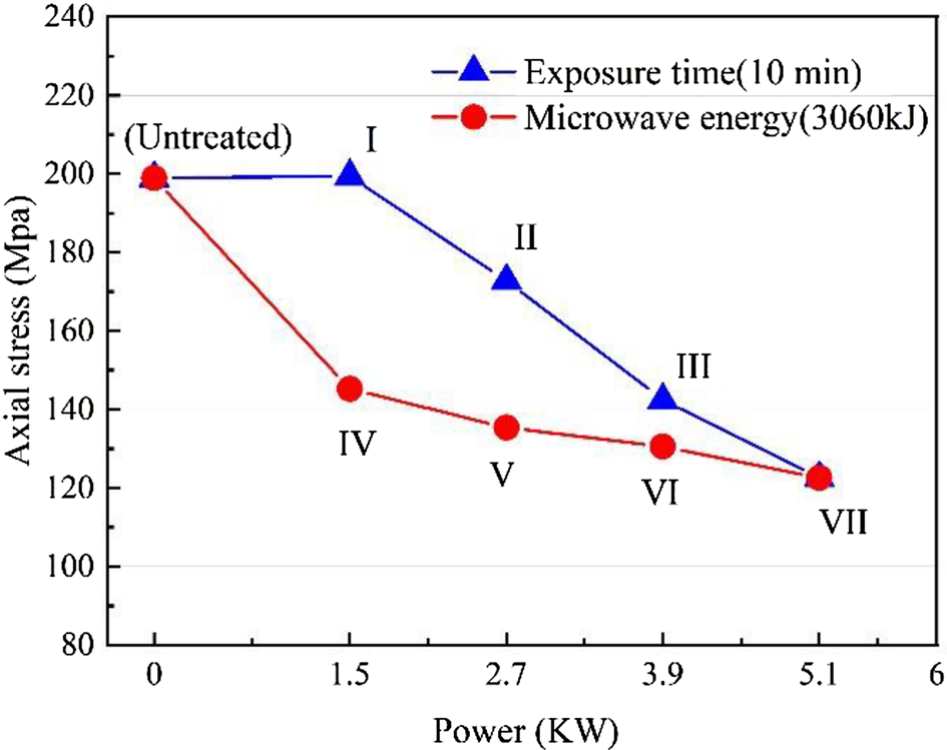
Relationship between UCS of samples with microwave power level.

The variation curve of UCS with power level for the basalt specimen is shown in Fig. 9. The UCS of the untreated basalt samples is 198. 91 MPa. Under the same irradiation time (t = 10 min), the UCS of the samples shows a nearly linear reduction with the increasing power level, which is about − 0.2%, 13.2%, 28.4%, and 46.4%, respectively. Under the same microwave energy (3060 kJ), the UCS of the basalt samples decreased significantly with increasing power levels, which are about 27.0%, 32.0%, 34.4%, and 46.4%, respectively. Under the same power level, the UCS of the samples decreases with the increase of microwave radiation time. However, the UCS of microwave-treated basalt samples with a power of 1.5 kW is 199.39 MPa and that increased by 0.2% compared to the untreated basalt samples.

### Correlation of AE data and nephogram of maximum principal strain field with stress–time curve

**Figure 10:**
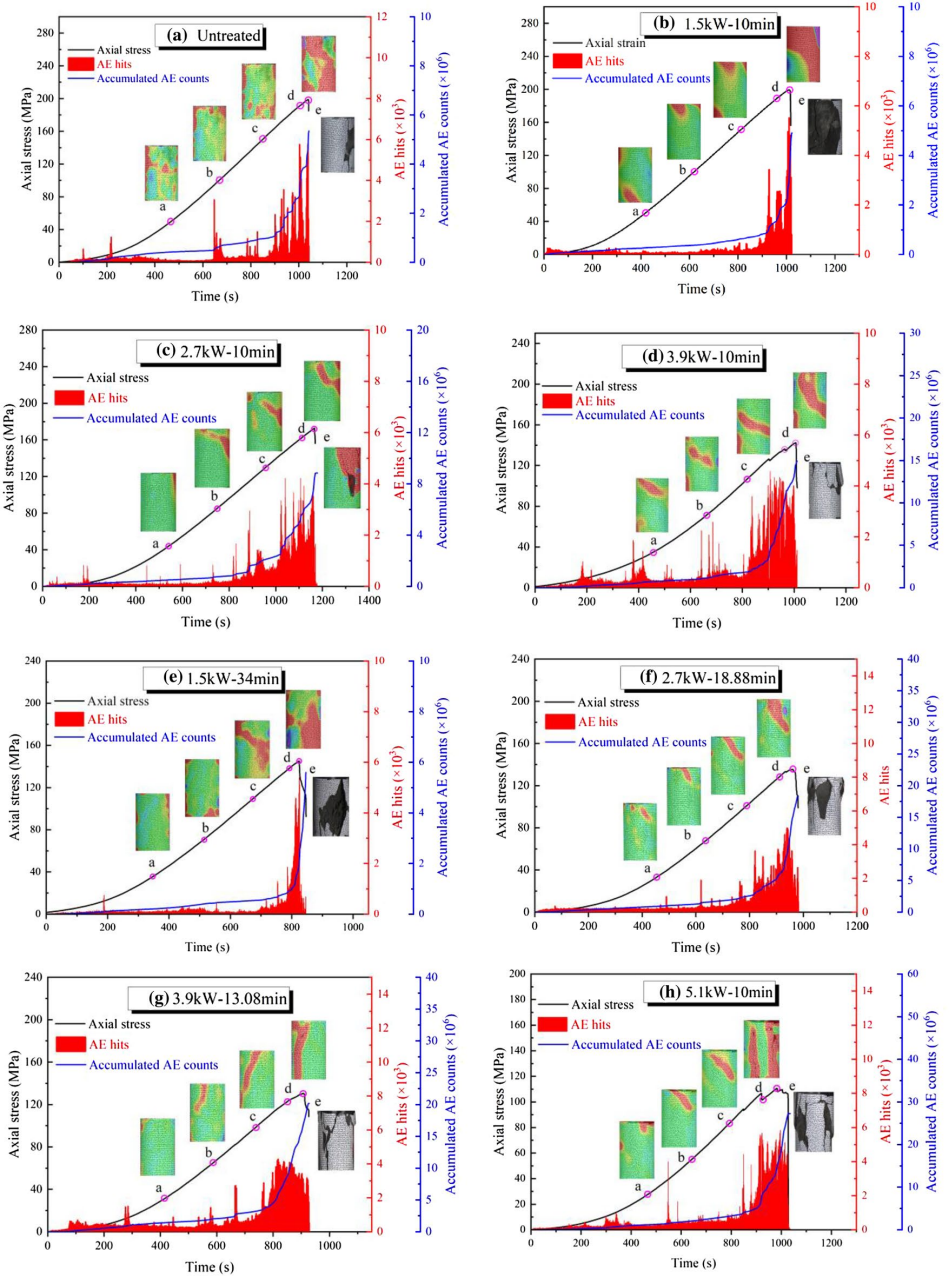
AE characteristics and maximum principal strain evolution of basalt samples under different exposure times and power:  (**a**) Untreated; (**b**) at 1.5kW for 10 min; (**c**) at 2.7kW for 10 min; (**d**) at 3.9 kW for 10 min; (**e**) at 1.5kW for 34 min; (**f**) at 2.7 kW for 18.88 min; (**g**) at 3.9 kW for 13.08 min; (**h**) at 5.1 kW for 10 min.

AE activity is caused by grain boundary movement, dislocation or closure, initiation, and propagation within a mineral particle or mineral grains^44,45^. Figure 10 shows the evolution of the maximum principal strain field and the AE characteristics of rocks under different exposure times and the power of microwave irradiation. The evolution of AE hits and cumulative AE counts correlated well with the stress-time curves. The points ’a’, ’b’, ’c’, and ’d’ in Fig. 8 represent 0.25, 0.5, 0.75, and 1 time of uniaxial compressive strength (UCS), respectively.

In Fig. 10a, during the ’oa’ phase (initial loading phase), the AE signals are few and the nephogram of the maximum principal strain field is randomly distributed, which is caused by the closure of pre-existing microcracks. But the samples by low-power treatment showed weak AE activity (Fig. 10b). In the ‘oa’ phase of Fig. 10c,d,h, the activity of AE hits and cumulative AE counts become intense as the power level increases. When enters the ’ab’ phase, weak AE signals continue to occur, cumulative AE events increase slowly, and the crack starts to expand. As the power level increases, strain concentration is generated in the upper part of the rock. Before point ‘c’ in Fig. 10, AE events grow steadily, the crack starts to expand and the intensity continues to increase. When the intensity reaches 0.75 UCS, the strain concentration region in the upper part of the rock gradually becomes clear. When entering the ‘cd’ stage, the crack expands rapidly and the AE signal intensity increases suddenly, which is in the rapid crack growth period. At this stage, the strain concentration region at both ends of the untreated specimens begins to form initially, expand and slowly connect diagonally along the sample. Taking Fig. 10h as an example, the maximum principal strain region in the upper part of the specimen gradually widens and moves downward, eventually forming a vertical strain region. Starting from point ‘c’, the strain concentration region in the upper part of the specimen develops rapidly, and the specimen fails when the peak stress is reached.

In addition, the activity of both AE hits and cumulative AE counts of rock specimens became more intense with increasing irradiation time at the same power level. At the same microwave energy (3060 kJ), the AE hits and cumulative AE counts of the samples are more intense with increasing power levels. This indicates that high power levels of microwave radiation can cause more damage to rocks.

### Changes in b-value during loading

The dynamic characteristics of b-value reflect the distribution characteristics of microfracture scales within the rock during the loading process. A larger b-value indicates that small-scale microfractures are dominant, while a smaller b-value indicates that large-scale microfractures occupy a larger proportion. Furthermore, the sudden change of b-value is one of the important precursors of rock failure. The b-value is defined in seismology by the classical Gutenberg-Richter law^46^

2 $$\mathrm{log}N=a-bM$$

where N is the total number of earthquakes of magnitude, M is the amplitude of the earthquake on the Richter scale (which is a logarithmic scale), a is an empirical constant, and b is the b-value. In the analysis of AE parameters, the amplitude can be divided by 20 to represent the AE magnitude M, i.e. M = A/20.

**Figure 11:**
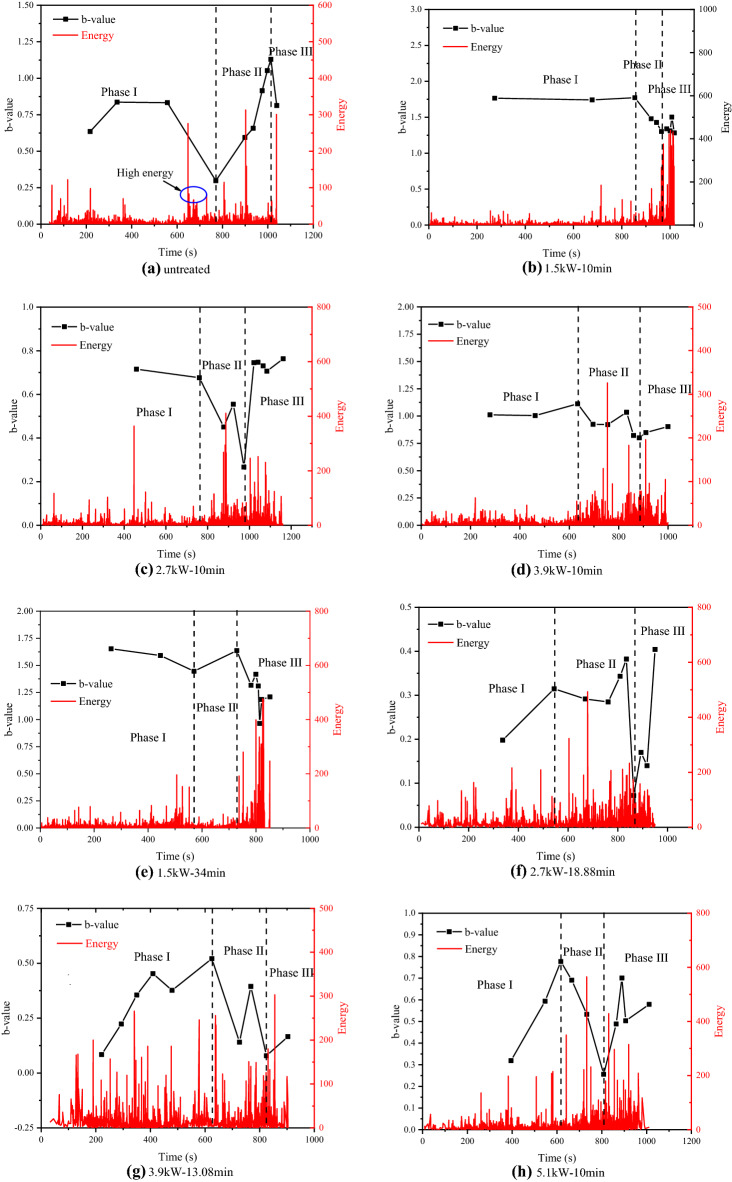
Changes in b-values for various times and various microwave irradiation energies:  (**a**) Untreated; (**b**) at 1.5kW for 10 min; (**c**) at 2.7kW for 10 min; (**d**) at 3.9 kW for 10 min; (**e**) at 1.5kW for 34 min; (**f**) at 2.7 kW for 18.88 min; (**g**) at 3.9 kW for 13.08 min; (**h**) at 5.1 kW for 10 min.

According to the degree of fluctuation of the b-value during loading, the whole rock specimen loading process under uniaxial compression is divided into three phases. At phase I, the curve of the b-value of the untreated basalt samples shows a convex shape. When loaded to 648 s, the b-value decreases, accompanied by a high energy signal release, indicating the formation of large-scale microfractures in the rock (Fig. 11a), which is verified interactively with Fig. 10a. Under the same radiation time, the b-value of the treated rock samples remains stable at phase I (as shown in Fig. 11b–d). Under the same microwave energy (3060 kJ), the b-value of the treated samples showed an upward trend at phase I in Fig. 11e–h, which is mainly characterized by medium-small-scale microcracks. At phase II, the b-value of the untreated samples gradually increases, indicating that the proportion of small-scale microfractures within the rock increases, which corresponds to the stable extension stage of microfractures. However, the b-value of the treated samples decreases with time and the AE energy increases with power level, which indicates that the proportion of large-scale microcracks in the rock increase. Entering phase III, the change of b value means that the rock is about to fail, which is an important precursor phenomenon. At the phase, the b-value of the untreated rock samples decreases rapidly, while the b-value of the treated sample gradually increases and the AE energy becomes active. It indicates that the proportion of large-scale cracks in untreated rocks increases, while the proportion of small and medium-sized microcracks in treated rocks increases.

### AE source location

**Figure 12:**
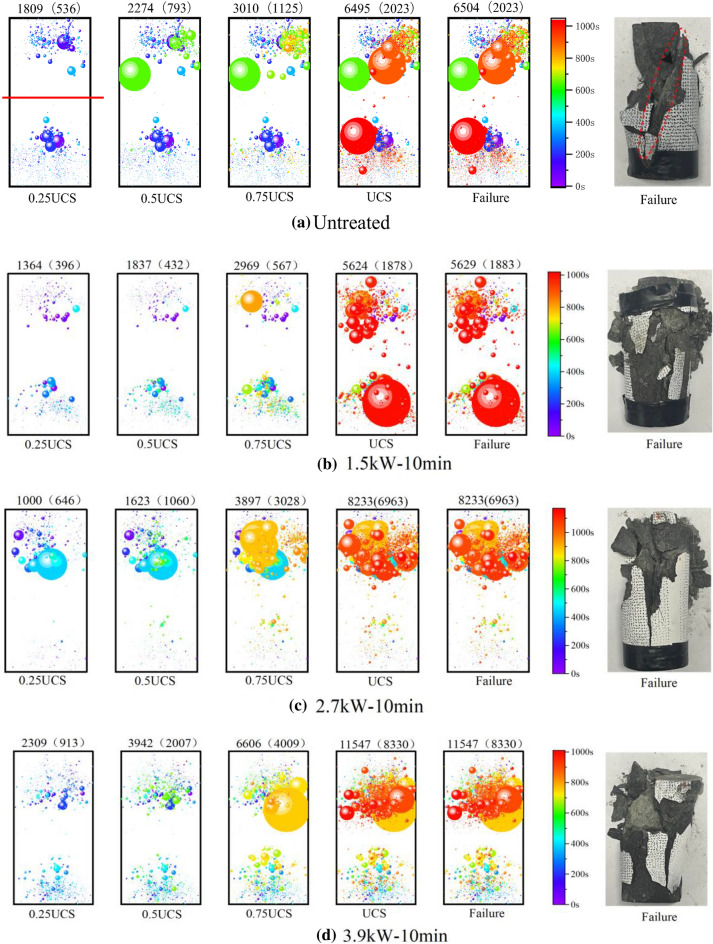

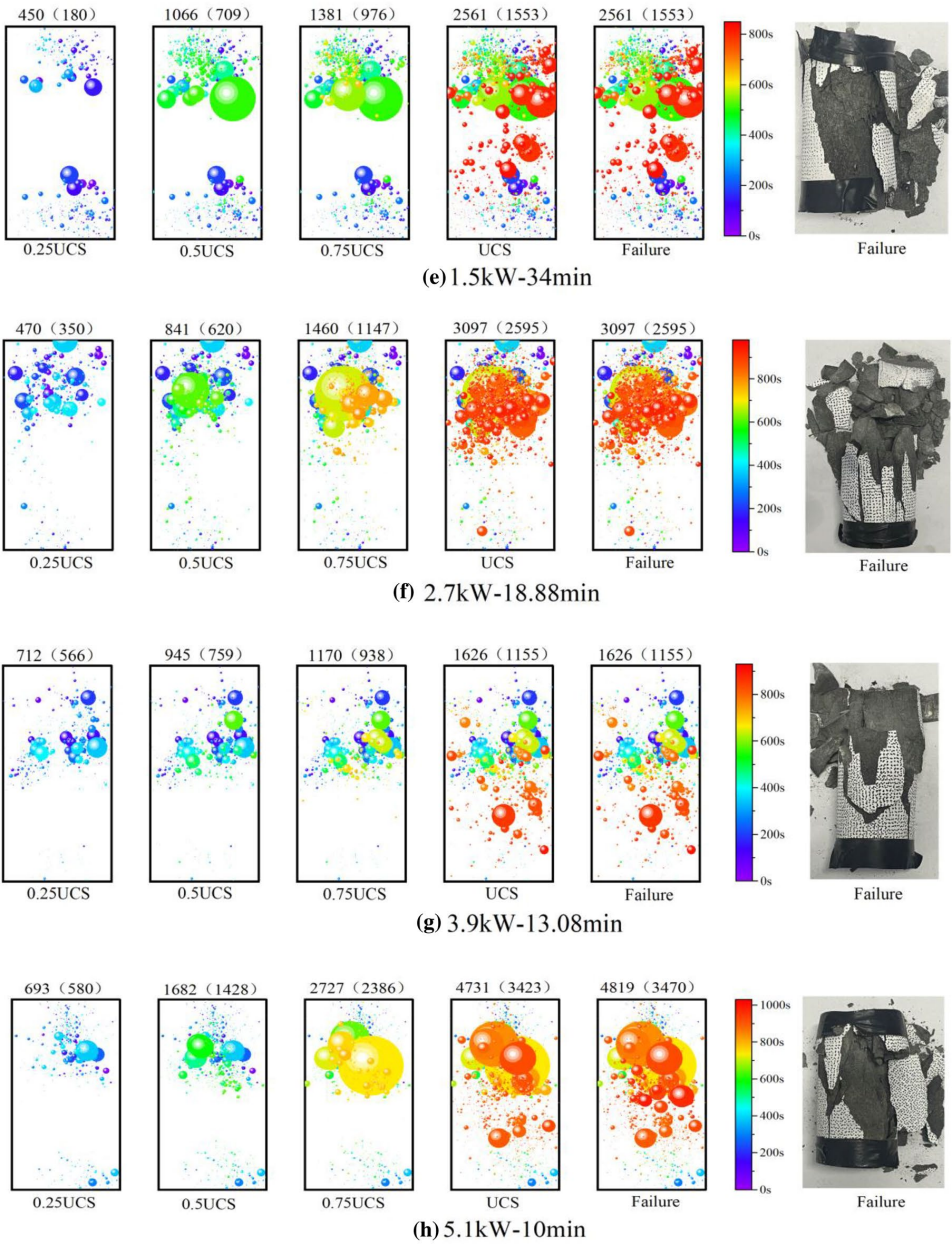
Spatial and temporal evolution of AE events in basalt samples:  (**a**) Untreated; (**b**) at 1.5kW for 10 min; (**c**) at 2.7kW for 10 min; (**d**) at 3.9 kW for 10 min; (**e**) at 1.5kW for 34 min; (**f**) at 2.7 kW for 18.88 min; (**g**) at 3.9 kW for 13.08 min; (**h**) at 5.1 kW for 10 min.

The AE source localization represents the space–time evolution law of crack propagation during loading. The signal intensity of the AE event reflects the severity of damage to the specimen in the uniaxial compression test. Figure 12 shows the spatial and temporal evolution of AE events (> 40 dB) at 0. 25, 0. 5, 0. 75, and 1 of uniaxial compressive strength (UCS). The diameter of the circle is proportional to the size of the signal strength of the AE event and the color of the circle reflects the time of appearance of the AE event. The number marked on the upper end of the cylindrical specimen represents the number of AE events. For example, 1809 in Fig. 12a represents the total number of AE events and 536 represents the number of AE events at the upper end of the sample (the upper and lower end of the sample is separated by a red line indicating the theoretical penetration depth of the microwave).

The number of AE events gradually increases with the stress in Fig. 12. The spatial distribution of AE events is usually disordered under axial stress (e. g., 0.25UCS, and 0.5UCS) in Fig. 12a, which is the result of the formation of new cracks in the rock and the closure of the initial microcracks. When the rock fails, there are 2023 AE events in the upper right corner of the rock, while 4472 AE events are in the lower right corner, and eventually, form an oblique failure plane. The result is consistent with the observed in the test, showing a shear failure mode (The dotted red ellipse is shown in Fig. 12a). The AE events of the treated samples are mainly concentrated in the upper part of the samples, which is because the microwave energy absorbed by the sample decreases exponentially with depth, leading to more thermal damage in the upper part of the rock (as shown in Fig. 12b–h). With the increase of axial stress, the AE events become denser at the upper end of the sample increases. Finally, a macroscopic failure plane is formed at the upper end, representing multi-fracture failure mode. The results show that the spatial distribution of AE events is consistent with the mode of failure observed in the experiment.

In the middle and early stages of load, the upper part and the lower part of the rock show different responses to stress. In Fig. 12d (0.25–0.5 UCS), the magnitude of AE events in the lower part of the rock is small, while a large yellow amplitude circle appears at 0.75 UCS. The results show that the upper part of the rock forms macroscopic cracks through compaction, expansion, development, and penetration, but the lower part of the rock at the corresponding time only has the initiation, development, and expansion of microcracks, and no effective macroscopic cracks are formed. At a higher stress level (greater than 80%), a large number of red amplitude circles form in the upper part of the rock, it can be found that the macrocracks and microcracks of the rock begin to extend downward and connect with the microcracks at the lower end, eventually leading to rock failure in Fig. 10d.

## Discussions

The behavior of surface temperature change of rock samples is directly related to the attenuation of waves in dielectric materials. When the wave propagates to the basalt sample, the microwave energy is absorbed by the microwave-absorbing mineral particles in rock, and the amplitude of the wave gradually decreases. If the reflected waves in the rock medium are ignored, the power density (the absorbed energy) in the rock decreases with depth, which results in a decrease in temperature with depth, as shown in Fig. 6.

Rocks are made up of many different minerals which have different abilities to absorb microwaves. During microwave radiation, the microwave-sensitive minerals in the rock absorb the microwaves, leading to an increase in the temperature of the rock. These minerals exhibit different physical and mechanical properties, resulting in anisotropy and nonuniformity due to thermal expansion^47,48^. When the thermal stress increases to a certain extent, transgranular cracks may occur in the rock mineral particles or intergranular cracks occur at the boundary of rock mineral particles. These transgranular cracks and intergranular cracks develop, expand, penetrate and finally form macroscopic cracks under the continuous action of microwave (Fig. 7).

**Figure 13:**
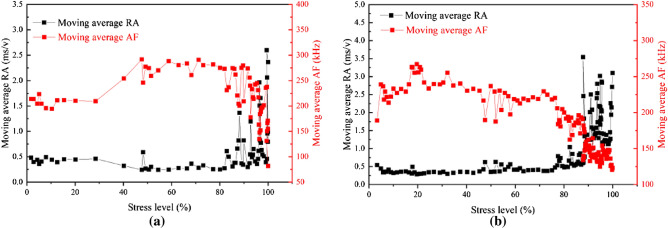
Changes in moving average RA and AF values during the loading. (**a**) Untreated; (**b**) 3.9 kW–10 min.

For unheated samples, high AF values and low RA values indicate that tensile cracks are predominant in the rock in Fig. 13a ^49–51^. When the local stress transcends the strength of the rock, the microcracks will cross the mineral particle boundary and form shear cracks^52^, resulting in rapid rock failure. As shown in Fig. 11a,b-value suddenly changes before the impending failure of rock, the proportion of large-scale microcracks gradually increases and develops fast relatively. The AE events of the untreated samples are randomly distributed, while the AE events of the treated samples are mainly concentrated in the upper part in Fig. 12a. For example, in Fig. 12d, at 0. 25, 0. 5, 0. 75, and 1 UCS, the percentage of AE events in the upper part accounted for the total AE events is 39. 54%, 50. 91%, 60. 69%, and 72. 14%, respectively. Microwaves cause a large number of thermal cracks in the upper part of the rock, resulting in a reduction in rock strength. At the same power level, with the increase of irradiation time, the degree of thermal damage of basalt increases, and the width and depth of fracture increase significantly in Fig. 7. In Fig. 13b, high RA and low AF values indicate that the proportion of tensile cracks increases, indicating that more thermal cracks participate in the failure process during the loading process. At low power levels, b-value fluctuates within a certain range, and new microfractures are rarely generated in phase I. When the power level exceeds 1.5 kW, the b-value fluctuation rule is roughly the same, that is, small-scale microfractures are dominated in phase I, large-scale microfractures in phase II, and small-scale microfractures in phase III. The failure process of untreated samples is relatively rapid, and b-value suddenly changes before the forthcoming destruction of rock. The catastrophe point of b-value of treated samples appeared earlier than that of untreated samples, which means that the rock has reached the precursor of failure earlier. Macroscopically, the tension cracks and shear cracks generate together, the debris increases obviously, the massive rock mass spalls off, and finally, multi-fracture failure occurs. However, the lower part of the rock is mostly broken in the form of splitting, and the rock fragments are relatively intact, indicating that the damage caused by microwave radiation to the upper part of the rock is more obvious. With the increase of power level, rock damage is more obvious. Therefore, the combination of high power level and short irradiation time can achieve the purpose of energy saving.

## Conclusion

To simulate rock excavation in a practical application scenario, the copper-foil-wrapping method is used in this study. After heating the basalt, the maximum surface temperature of basalt samples increases with the increase of power level. Under the same power level, the maximum surface temperature of basalt specimens increases with the increase of radiation time. High power levels and a long time of microwave irradiation cause more damage to the rock, leading to a reduction in strength of basalt. During loading process, the AE source events of treated basalt are mainly distributed in the upper part of the rock. At low power levels, b-value fluctuates within a certain range, and new microfractures are rarely generated in phase I. When the power level exceeds 1.5 kW, the b-value fluctuation rule is roughly the same, and small-scale microcracks are dominant in the rock in phase III. The treated basalts reach the precursor of failure earlier than untreated. The failure mode of basalt changes from shear failure to multi-failure failure.

## Data Availability

All data, models, or code that support the findings of this study are available from the corresponding author (zytyut1@126.com) upon reasonable request.
